# Low serum calcium is associated with perioperative blood loss and transfusion rate in elderly patients with hip fracture: a retrospective study

**DOI:** 10.1186/s12891-021-04914-1

**Published:** 2021-12-07

**Authors:** Zhicong Wang, Xi Chen, Yan Chen, Ling Yang, Hong Wang, Wei Jiang, Shuping Liu, Yuehong Liu

**Affiliations:** Department of Orthopedic Surgery, Deyang People’s Hospital, Orthopaedic Center of Deyang City, Deyang, 618000 Sichuan China

**Keywords:** Hypocalcemia, Hip fracture, Elderly, Blood loss, Blood transfusion

## Abstract

**Background:**

To investigate whether hypocalcemia influenced total blood loss and transfusion rate in elderly patients with hip fracture.

**Methods:**

From our hip fracture database, patients were consecutively included between January 2014 and December 2020. Serum calcium level was corrected for albumin concentration, and hypocalcaemia was defined as corrected calcium < 2.11 mmol/L. Hemoglobin and hematocrit were obtained on admission day and postoperative day, and blood transfusions were collected. According to the combination formulas of Nadler and Gross, the total blood loss of each patient was calculated. Risk factors were further analyzed by multivariate linear regression.

**Results:**

A total of 583 consecutive elderly hip fracture patients were finally included (mean age 79.32 ± 8.18 years, 68.61% female). On admission, the mean serum corrected calcium level was 2.17 ± 0.14 mmol/L, and the prevalence of hypocalcemia was 33.11% (95% *CI*: 29.42–37.02). When comparing patients with normal calcium, hypocalcemia patients exhibited a higher blood transfusion rate (7.69% vs 16.06%, *P* < 0.05), and significantly larger total blood loss (607.86 ± 497.07 ml vs 719.18 ± 569.98 ml, *P* < 0.05). Multivariate linear regression analysis showed that male, anemia on admission, time from injury to hospital, intertrochanteric fracture, blood transfusion and hypocalcemia were independently associated with increased total blood loss (*P* < 0.05).

**Conclusion:**

Hypocalcemia is common in elderly patients with hip fracture, and significantly associated with more total blood loss and blood transfusion. The other risk factors for increased total blood loss are male, anemia on admission, time from injury to hospital, intertrochanteric fracture, and blood transfusion.

**Level of evidence:**

Level III, retrospective study.

## Background

With an increasing aging population, hip fractures become a major public health issue, along with high morbidity, disability, mortality, and social costs [1, 2]. Even more, it is estimated that there will be 4.5 million hip fractures worldwide by the year 2050, and 1.3 million cases will be in China [3, 4]. After the initial injury, hip fracture patients may suffer from a large amount of total blood loss, which vary from 859 ml to 1208 ml in patients with femur neck fracture [5–7], and 406 ml to 1269 ml in patients with intertrochanteric fracture [6, 8–11]. Similarly, a prospective study found that the blood loss can reach up to 1013 ml even prior to the surgery [12].

Blood loss in elderly patients makes them prone to perioperative anemia due to the presence of comorbidities and limited physiological reserve [6]. Recently, an increasing number of studies have confirmed that low hemoglobin or anemia on admission was significantly associated with increased mortality, postoperative complications, and poorer physical function [13–15]. As we known, blood transfusion is the most commonly used intervention to correct anemia, but causes more adverse events in elderly patients [16].

Serum calcium as the coagulation factor IV, participates in the regulation of coagulation cascade [17]. Hypocalcemia is a common electrolyte disorder in hospitalized patients, nearly 56.2% in trauma patients [18]. Many studies have already investigated its harmful effect, and found that hypocalcemia was associated with more bleeding or blood transfusion in patients with intracerebral hemorrhage [19], postpartum hemorrhage [20], upper gastrointestinal bleeding [21], shocked trauma [22], and total knee arthroplasty [23].

Several studies indicated that the prevalence of hypocalcemia was common in elderly orthopedic patients [23–25]. For this reason, we hypothesized that low serum calcium on admission may led to more perioperative blood loss. To our knowledge, there is no available literature regarding this issue in hip fracture patients undergoing surgery. Therefore, we aimed to close this gap and investigate whether serum calcium influenced total blood loss and transfusion rate in these patients.

## Methods

### Study design and population

**Fig. 1:**
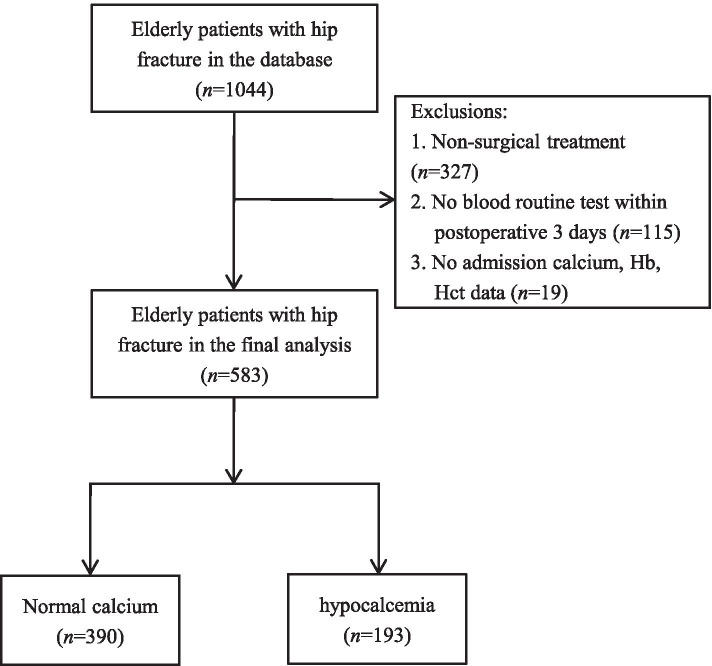
Flow chart of patient inclusion

We performed a retrospective study using data from our hip fracture database, which has been described in detail previously [26]. From January 2014 to December 2020, patients were enrolled consecutively into the database when the following criteria were met: (1) confirmed diagnosis of hip fracture, but not pathological fracture; (2) age ≥ 60 years; (3) caused by low-energy fall from a standing height or less; (4) fresh fracture less than 3 weeks. In this study, patients without surgical treatment (*n* = 327), blood routine test within postoperative 3 days (*n* = 115), as well as admission calcium, hemoglobin (Hb), hematocrit (Hct) data (*n* = 19) were excluded (Fig. 1). The study protocol was approved by the Institutional Ethics Committee at Deyang People’s Hospital (IRD number 2021–04-019-K01). Permission to waive the informed consent was obtained from the institutional review board for this observational retrospective study.

### Data collection procedures

The following data were extracted from this hip fracture database, including demographics (age, sex, height, weight), medical history (hypertension, diabetes mellitus), fracture information (time from injury to hospital, hip fracture type), and laboratory tests (calcium, albumin, Hb, Hct). Also, charlson comorbidity index (CCI) was calculated for each patient based on 17 comorbidities, and categorized as none (CCI = 0), low (CCI = 1), or moderate/severe (CCI ≥ 2) [27]. Body mass index (BMI) was calculated as BMI = Weight (kg)/Height (m)^2^. Time from injury to hospital was defined as the interval between the injury date and the subsequent admission date. Hip fracture type was confirmed by X-ray and/or computed tomography (CT), and then classified as femur neck or intertrochanteric fracture. According to the WHO criteria, anemia was defined as Hb < 130 g/dl for men and < 120 g/dl for women. Owing to the influence of albumin concentration, serum calcium was corrected using the following formula: corrected calcium = measured serum calcium (mmol/L) + 0.02 × [40.0 – albumin (g/L)] [28]. Using the normal reference range in our hospital (2.11–2.52 mmol/L), hypocalcemia was defined as corrected calcium < 2.11 mmol/L, and patients were further grouped into normal calcium group and hypocalcemia group.

Moreover, we collected the requirement of blood transfusion and transfusion volume from medical records. Blood transfusion at our institution was performed only when an absolute Hb < 80 g/L independent of signs or symptoms of anemia, or Hb < 90 g/L for symptomatic patients (extreme weakness, chest pain, extreme paleness, or major bleeding) with destabilizing vital signs (heart rate > 100 beats/min or systolic blood pressure < 90 mmHg).

### Total blood loss calculation

The patient blood volume (BV) was estimated according to the Nadler formula [29]: BV (L) for men = height (m)3 × 0.367 + weight (Kg) × 0.032 + 0.604, BV (L) for women = height (m)^3^ × 0.356 + weight (Kg) × 0.033 + 0.183. The total blood loss (TBL) was calculated according to the Gross formula [30]: TBL (ml) = BV (L) × (Hct_adm_ – Hct_post_)/Hct_ave_ × 1000, where Hct_adm_ is the initial admission Hct, Hct_post_ is Hct within 3 days after surgery, and Hct_ave_ is the average of Hct_adm_ and Hct_post_. When transfusion was performed from the time of admission to the data of postoperative blood routine test, TBL (ml) = BV (L) × (Hct_adm_ – Hct_post_)/Hct_ave_ × 1000 + blood transfusion (ml). A unit of red blood cell transfusion is approximately 200 ml.

### Statistical analysis

Continuous variables were described as mean ± standard deviation (SD), and analyzed with Student’s t-test, while categorical variables were expressed as frequency (percentage), and compared with the chi-squared test. Linear regression analysis was used to test the correlation of various clinical factors with perioperative total blood loss. Variables with *P* < 0.1 were included in multivariate linear regression analysis to identify the independent risk factors associated with total blood loss. All reported *P* values are two-sided, and *P* < 0.05 were considered statistically significant. All analyses were performed using JMP Pro software (version 16.0; SAS Institute Inc., Cary, NC, USA).

## Results

### Patient characteristics

A total of 583 consecutive elderly patients with hip fracture were included in this study. The baseline characteristics are shown in Table 1. On admission, the mean corrected calcium was 2.17 ± 0.14 mmol/L, and the prevalence of hypocalcemia was 33.11% (95% *CI:* 29.42–37.02). When comparing patients with normal calcium, hypocalcemia patients had a higher incidence of anemia (79.79% vs 67.95%, *P* < 0.05). Apart from this, no significant differences were observed between the two groups.

**Table 1:** Baseline characteristics according to serum calcium

Variables	Normal calcium (***n*** = 390)	Hypocalcemia (***n*** = 193)	***P value***
Age (years)	78.96 ± 8.45	80.04 ± 7.59	0.135
Sex, n (%)			0.076
Male	113 (28.97)	70 (36.27)	
Female	277 (71.03)	123 (63.73)	
Height (cm)	157.44 ± 6.59	158.11 ± 6.15	0.243
Weight (Kg)	54.00 ± 8.97	53.87 ± 6.98	0.861
BMI (Kg/m^2^)	21.72 ± 2.90	21.51 ± 2.18	0.372
Hypertension, n (%)	142 (36.41)	67 (34.72)	0.688
Diabetes mellitus, n (%)	70 (17.95)	40 (20.73)	0.423
Anemia on admission, n (%)	265 (67.95)	154 (79.79)	0.002
CCI score, n (%)			0.377
None	213 (54.62)	99 (51.30)	
Low	120 (30.77)	57 (29.53)	
Moderate/severe	57 (14.62)	37 (19.17)	
Time from injury to hospital (h)	9.5 (3.0, 48.0)	10.0 (2.0, 72.0)	0.624
Hip fracture type, n (%)			0.769
Femoral neck	195 (50.00)	94 (48.70)	
Intertrochanteric	195 (50.00)	99 (51.30)	

*Abbreviations*: *BMI* Body mass index, *CCI* Charlson comorbidity index

### Perioperative blood loss

The perioperative Hb, Hct, transfusion and blood loss data are presented in Table 2. There were significant differences in admission and postoperative Hb and Hct levels between the two groups (*P* < 0.05). Moreover, the drop in Hb for hypocalcemia and normal calcium patients were 17.25 ± 15.16 g/L and 12.24 ± 17.39 g/L, and the difference was statistically significant (*P* < 0.05). Likewise, the difference in Hct was significantly larger in hypocalcemia group compared with normal calcium group (5.22 ± 4.52% vs 3.72 ± 5.40%, *P* < 0.05). Also, the blood transfusion rate in hypocalcemia group was higher than normal calcium group (16.06% vs 7.69%, *P* < 0.05), but transfusion volume difference did not reach significance (*P* = 0.533). The mean total blood loss for hypocalcemia group was 719.18 ± 569.98 ml, which was significantly larger than normal calcium group (607.86 ± 497.07 ml, *P* < 0.05).

**Table 2:** Perioperative hemoglobin, hematocrit, transfusion and blood loss according to serum calcium

Variables	Normal calcium (***n*** = 390)	Hypocalcemia (***n*** = 193)	***P value***
Blood volume (L)	3.48 ± 0.55	3.52 ± 0.53	0.373
Hb (g/L)			
Admission	113.25 ± 18.58	103.92 ± 20.22	< 0.001
Postoperative	101.01 ± 16.82	86.67 ± 15.99	0.003
△Hb	12.24 ± 17.39	17.25 ± 15.16	< 0.001
Hct (%)			
Admission	35.09 ± 5.34	32.34 ± 5.93	< 0.001
Postoperative	31.37 ± 5.07	27.12 ± 4.70	0.004
△Hct	3.72 ± 5.40	5.22 ± 4.52	0.001
Blood transfusion, n (%)	30 (7.69)	31 (16.06)	0.003
Mean transfusion volume (ml)	516.67 ± 219.85	558.06 ± 289.57	0.533
Mean total blood loss (ml)	607.86 ± 497.07	719.18 ± 569.98	0.016

*Note*: △Hb, △HCT was difference from admission to postoperative

*Abbreviations*: *Hb* Hemoglobin, *Hct* Hematocrit

### Factors influencing total blood loss

Linear regression analysis showed that BMI, anemia on admission, time from injury to hospital, hip fracture type, blood transfusion and serum calcium were associated with total blood loss (Table 3). In the multivariate linear regression analysis (Table 4), factors that were independently associated with increased total blood loss were male, anemia on admission, time from injury to hospital, intertrochanteric fracture, blood transfusion and hypocalcemia (*P* < 0.05).

**Table 3:** Linear regression analysis of factors associated with total blood loss

Variable	R^**2**^	***P*** value
Age (years)	0.002	0.237
Sex	0.005	0.076
BMI (Kg/m^2^)	0.009	0.025
Hypertension	0.001	0.384
Diabetes mellitus	0.002	0.313
Anemia on admission	0.007	0.045
CCI score	0.001	0.662
Time from injury to hospital (h)	0.015	0.003
Hip fracture type	0.044	< 0.001
Blood transfusion	0.031	< 0.001
Serum calcium	0.010	0.016

**Table 4:** Multivariate linear regression analysis of factors associated with total blood loss

Variable	Coefficient B	SE	95% CI for B	***P*** value
Constant	563.362	178.579	212.615–914.109	0.002
Sex (male/female)	53.183	22.211	9.559–96.806	0.017
BMI (Kg/m^2^)	14.083	7.907	−1.446-29.613	0.075
Anemia on admission	107.230	25.027	58.073–156.386	< 0.001
Time from injury to hospital (h)	−0.552	0.231	−1.006 - -0.098	0.017
Type of fracture (intertrochanteric/femoral neck)	126.319	21.644	83.809–168.830	< 0.001
Blood transfusion	143.271	34.274	75.955–210.588	< 0.001
Serum calcium (hypocalcemia/normal)	50.338	22.054	7.022–93.655	0.023

*Abbreviations*: *SE* Standard error, *CI* Confidence interval

## Discussion

In this study, the prevalence of hypocalcemia was 33.11% in hip fracture patients, which was similar to other studies in elderly orthopedic patients [23–25]. Similarly, almost 27.72% of hospital patients may experience hypocalcemia, and the incidence was the highest in patients over 65 years [31]. Regarding the harm of hypocalcemia, a recent review summarized the relationships between preoperative hypocalcemia and postoperative adverse complications in elderly patients [32], and another systematic review found admission hypocalcemia was associated with increased mortality in trauma patients [18]. These findings suggest that clinicians should pay attention to hypocalcemia because of life-threatening consequence [31].

Beyond those mentioned above, serum calcium is a key component of coagulation cascade. Hence, we hypothesized that low serum calcium may led to more perioperative blood loss. As expected, we found that hypocalcemia patients had more total blood loss in elderly patients with hip fracture, along with larger differences in Hb and Hct levels from admission to postoperative day. Even after adjusting for potential confounding factors, hypocalcemia still played an independent role in blood loss, and led to an almost 50.338 ml increase in total blood loss. Consistent with this finding, Morotti et al. [19] reported that the presence of hypocalcemia on admission was indeed associated with the extent of bleeding in patients with intracerebral hemorrhage. This effect was also observed in patients with postpartum hemorrhage [20], and total knee arthroplasty [23]. For this reason, trials are needed to assess whether correction of hypocalcemia will lead to decreased blood loss [20, 21].

In this study, the rate of blood transfusion was 10.46% (95% *CI*: 8.23–13.21), which was lower than report of Nikolaou et al. (63.47%) [6], Ohmori et al. (32.67%) [33], and Guo et al. (29.7%) [5] in hip fracture patients. The reason might be that the blood loss continue until several days after surgery [9], but this study only analyzed the blood transfusions from admission to the time of blood routine test. Consistent with the finding of total blood loss, hip fracture patients with hypocalcemia exhibited a higher blood transfusion rate, although transfusion volume difference did not reach significance. Also, trauma patients [22], and upper gastrointestinal bleeding patients [21] with hypocalcemia were most likely to receive a blood transfusion.

Moreover, we found some other factors associated with increased total blood loss, including male, time from injury to hospital, intertrochanteric fracture, and blood transfusion, which were consistent with previous studies [5, 10, 11]. In this study, anemia on admission was identified as another independent risk factor for total blood loss. Similarly, Miguel et al. [34] showed that anemia on admission was associated with more severe intracerebral hemorrhage. Also, low Hb level was associated with blood transfusion in elderly patients with hip fracture [13], this may be the reason for the increased total blood loss.

However, this study has some limitations. First, as this was a retrospective study, we were not able to collect sufficient information on history of osteoporosis and anti-osteoporotic agents, which may affect serum calcium level. In particular, we also did not obtain the anticoagulant and antiplatelet treatment. Yet, a recent study found antiplatelet treatment did not affect perioperative blood loss in patients with hip fracture [33]. Second, it was a single-center study, and the sample size was small. Therefore, multi-center and larger-scale studies are needed to confirm our results. Third, we only used serum albumin corrected calcium instead of ionized calcium to evaluate the true level of calcium, which may be inaccurate in the presence of protein and pH imbalances. Finally, the fluid volume administered perioperatively was not controlled, and this may affect the calculation of the total blood loss. Likewise, the formula to estimate blood loss does not represent the accurate amount of blood loss [33].

## Conclusion

Hypocalcemia is common in elderly patients with hip fracture, and significantly associated with more total blood loss and blood transfusion. The other risk factors for increased total blood loss are male, anemia on admission, time from injury to hospital, intertrochanteric fracture, and blood transfusion.

## Data Availability

The data used during the current study are available from the corresponding author on reasonable request.

## Abbreviations

Hb: Hemoglobin
Hct: Hematocrit
CCI: Charlson comorbidity index
BMI: Body mass index
CT: Computed tomography
BV: Blood volume
TBL: Total blood loss
SD: Standard deviation
